# Forecasting future Humphrey Visual Fields using deep learning

**DOI:** 10.1371/journal.pone.0214875

**Published:** 2019-04-05

**Authors:** Joanne C. Wen, Cecilia S. Lee, Pearse A. Keane, Sa Xiao, Ariel S. Rokem, Philip P. Chen, Yue Wu, Aaron Y. Lee

**Affiliations:** 1 Department of Ophthalmology, University of Washington, Seattle, WA, United States of America; 2 NIHR Biomedical Research Centre for Ophthalmology at Moorfields Eye Hospital, Moorfields Eye Hospital NHS Foundation Trust and University College London (UCL) Institute of Ophthalmology, London, United Kingdom; 3 eScience Institute, University of Washington, Seattle, WA, United States of America; Massachusetts Eye & Ear Infirmary, Harvard Medical School, UNITED STATES

## Abstract

**Purpose:**

To determine if deep learning networks could be trained to forecast future 24–2 Humphrey Visual Fields (HVFs).

**Methods:**

All data points from consecutive 24–2 HVFs from 1998 to 2018 were extracted from a university database. Ten-fold cross validation with a held out test set was used to develop the three main phases of model development: model architecture selection, dataset combination selection, and time-interval model training with transfer learning, to train a deep learning artificial neural network capable of generating a point-wise visual field prediction. The point-wise mean absolute error (PMAE) and difference in Mean Deviation (MD) between predicted and actual future HVF were calculated.

**Results:**

More than 1.7 million perimetry points were extracted to the hundredth decibel from 32,443 24–2 HVFs. The best performing model with 20 million trainable parameters, CascadeNet-5, was selected. The overall point-wise PMAE for the test set was 2.47 dB (95% CI: 2.45 dB to 2.48 dB), and deep learning showed a statistically significant improvement over linear models. The 100 fully trained models successfully predicted future HVFs in glaucomatous eyes up to 5.5 years in the future with a correlation of 0.92 between the MD of predicted and actual future HVF and an average difference of 0.41 dB.

**Conclusions:**

Using unfiltered real-world datasets, deep learning networks show the ability to not only learn spatio-temporal HVF changes but also to generate predictions for future HVFs up to 5.5 years, given only a single HVF.

## Introduction

Glaucoma is a leading cause of blindness worldwide.[1] Of the 32.4 million blind people globally in 2010, an estimated 4.0 to 15.5% were from glaucoma.[2] Visual field (VF) testing using standard automated perimetry remains a clinical standard for monitoring glaucomatous damage, and estimating future VF loss is integral to glaucoma management. With Humphrey standard automated perimetry, linear regression of global indices such as mean deviation (MD) and visual field index (VFI) are commonly used to assess glaucoma progression and risk of future progression.[3] However, as glaucomatous visual field changes are frequently focal, these global indices may fail to detect more subtle changes. Point-wise linear regression analyses were developed to evaluate changes at each test point and have been shown to identify glaucomatous progression earlier than global indices.[4] A number of linear and non-linear regression models have been described,[5–7] yet these models often assume a constant amount or rate of progression and typically evaluate progression rates of single visual field test points independent of adjacent test points. Furthermore, a relatively large number of VF tests are frequently required in these models to achieve reasonably accurate predictions.[7]

Recently, predictive machine learning methods have been developed with the advent of deep neural networks. Specifically the integration of "spatially aware" convolutional filters [8] paired with non-linear activation functions [9] has allowed computer vision researchers to achieve unprecedented accuracy in classification of real-world objects. These data-driven methods are able to directly ingest pixel intensities of a photograph and learn spatial features important for a given task. Ophthalmologic applications of deep learning include optical coherence tomography (OCT) image classification[10] and segmentation,[11–13] fundus photo analysis,[14] and the diagnosis and classification of glaucoma based on disc photos, OCTs and VFs.[15–20] While most studies using deep learning have focused on classification, there has been limited application of deep learning in forecasting future findings. In addition to classification, deep learning algorithms can include segmentation, regression, localization, and generative models. The purpose of this study is to apply a deep learning generative model to predict future VFs with preserved spatial information using minimal baseline VF test input.

## Materials and methods

This study was approved by the Institutional Review Board at the University of Washington. A waiver of consent and waiver of HIPAA authorization were granted by the IRB. All patients obtaining a Humphrey Visual Field (HVF) (Humphrey Visual Field Analyzer II-i, Carl Zeiss Meditec, Inc. Dublin, CA) using program 24–2 and the Swedish Interactive Thresholding Algorithm (SITA) Standard between the years of 1998 and 2018 at the University of Washington were included in the study. Using an in-house created, fully automated extraction algorithm that yields 54 perimetry point values to the hundredth decibel, consecutive extraction of HVF 24–2 data was performed. Additional clinical variables such as age, gender, date of HVF, and eye tested were extracted and fully de-identified prior to subsequent analysis.

**Fig 1:**
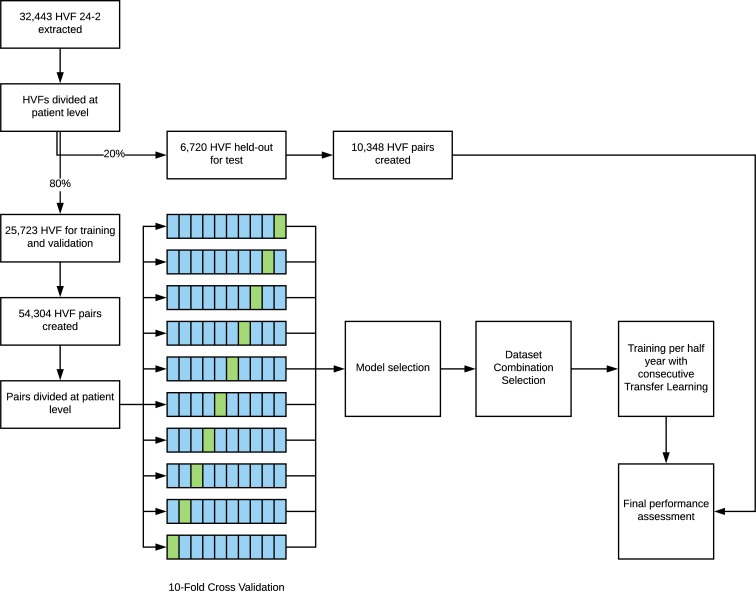
Flowchart of study design. A combination of cross validation and held-out test set was used for the development and evaluation of the models.

The resulting HVFs were then divided at the patient level into two parts: 80% for training and validation and 20% as a held-out test set. With the training and validation test, a total of 10 disjoint sets, divided again at the patient level, were created for 10-fold cross validation. For each set in the training, validation, and test sets, the HVFs were paired into every possible temporal combination, binned into 0.5 year intervals. Patients with only one visual field were excluded from the study. Any combinations greater than 5.5 years and less than 0.75 years in interval were excluded. For example, if there were three HVFs done: A, B, and C and the time interval between A and B was 3 months and the time interval between B and C was 1.5 years, we excluded the combination of A and B since this was less than 0.75 years. However, we included the combinations of A and C as well as B and C since each of these combinations were greater than 0.75 years. Three main phases of model development were then performed: model architecture selection, dataset combination selection, and time-interval model training with transfer learning. The final model's weights were then frozen at each time-interval and were used to assess the performance on the final held-out test set. The study design is depicted and summarized in Fig 1, and the evaluation strategy is shown in S1 Fig.

Model selection was performed by testing 9 different network architectures (Table 1). Deep learning models were created such that they could take an input tensor with the HVF as a single 8x9 tensor and output as a single 8x9 tensor representing the target HVF. The final layer activation was set to linear, and the loss function for each model was defined as masked point-wise mean absolute error (PMAE). The models were randomly initialized with parameters using the Xaiver algorithm[21] and optimized using Adam, a stochastic gradient descent optimization algorithm,[22] with an initial learning rate of 1 x 10^−3^. Each model was trained for 1000 epochs 10 times, and validation accuracy was assessed at the end of each epoch using the interval data at one year (0.75 year to 1.25 years). The highest validation accuracy was recorded for each model and 10-fold cross-validation. The best performing model was then carried forward to all the subsequent training.

**Table 1:** Different model architectures trained for 1,000 epochs.

	Layers (n)	Batch Size	Optimizer with learning rate	Space Complexity (MB)	Parameters
Fully Connected	2	32	Adam 1x10^-3^	4	336,968
FullBN-3	10	32	Adam 1x10^-3^	23	1,921,795
FullBN-5	16	32	Adam 1x10^-3^	40	3,472,771
FullBN-7	22	32	Adam 1x10^-3^	58	5,023,747
Residual-3	12	32	Adam 1x10^-3^	27	2,332,163
Residual-5	18	32	Adam 1x10^-3^	45	3,883,139
Residual-7	24	32	Adam 1x10^-3^	63	5,434,115
Cascade-3	10	32	Adam 1x10^-3^	81	6,992,086
Cascade-5	16	32	Adam 1x10^-3^	238	20,694,754

Clinical dataset combination selection was done by taking the best performing model in the previous step and testing every possible combination of clinical predictor variables. Categorical variables such as eye and gender were appended in a one-hot vector format to the input tensor and continuous variables were encoded as a single additional tensor face with every cell encoded as the continuous value. For example, the input tensor without any additional data is shaped 1x8x9 encoding the spatial information of the perimetry sensitivities. If age were added to the input, the tensor would become 2x8x9 with the first 8x9 encoding the perimetry sensitivities and the second 8x9 array with every cell value set to the age. If eye laterality were added to the input, the tensor would become 3x8x9 with the first 8x9 encoding perimetry values and the 2x8x9 encoding the eye laterality as a one-hot vector representation. Each data combination was again trained for 1000 epochs 10 times and validation accuracy was assessed at the end of each epoch using the interval data at one year (0.75 year to 1.25 years). The highest validation accuracy was recorded for each data combination and 10-fold cross-validation. The best performing data combination was then carried forwarded to the all the subsequent training.

After the best performing model and data combination was determined in the prior two phases, time intervals from 0.75 years to 5.5 years in 0.5 year intervals were trained. Transfer learning was performed with weights carried from the first temporal interval, and consecutive transfer learning was performed with weights carried from the immediately preceding time interval. Final accuracy and performance were assessed by predicting the final held-out test set. The mean predictions from the 10-fold cross validation were used to generate each prediction.

All deep learning was performed with Keras (version 2.1.2, https://keras.io/)[23] and Tensorflow (version 1.4.1, https://www.tensorflow.org/).[24] Custom code was written in Python (version 2.7.12, https://www.python.org/) to scale and automate the training, and all training was performed using a single server equipped with 8 x Nvidia Tesla P100 graphics processing units with NVIDIA cuda (v8.0) and cu-dnn (v6.0) libraries (http://www.nvidia.com). Statistical analyses were performed using R (version 3.4.3, https://www.r-project.org/). The code has been open sourced and is available on Github (https://github.com/uw-biomedical-ml/hvfProgression).

We compared the deep learning model against three baseline models in ascending order of complexity. The first model (simulated model) is based on the mean and standard deviation for the rate of progression (ROP) from the literature. Specifically, we used data from the Early Manifest Glaucoma Trial and used -0.36 dB / year and 0.60 dB / year for the mean and standard deviation for the rate of progression.[25] Mathematically, the rate of progression can be considered a Gaussian distribution model with a mean rate of progression of -0.36*t and standard deviation 0.60*t, where *t* is the time in years. For each point in an input HVF, we sampled from this normal distribution and calculated a point-wise prediction of the future HVF. In the second model (empirical ROP model), we measured the empirical ROP distribution from the training set and applied it in the same manner as the first model in the held-out test set. In the third model (regressed point-wise model), the training set was used to regress 54 different linear models, one for each perimetry point. This model was then applied to the test set to measure the performance. Further details are provided in the supplementary materials.

## Results

A total of 32,443 24–2 HVFs were extracted from 1998 to 2018 contiguously resulting in 1,751,922 perimetry points with hundredth decimal precision. All available HVFs were used, and no filtering or data cleaning was performed prior to the training of the models. The HVFs were split into training, validation, and held-out test sets as described in Fig 1. Table 2 shows the baseline demographic factors of the study population.

**Table 2:** Baseline demographic factors at patient eye level.

	Training / Validation Set	Held-out Test Set	Total
Patients (n)	3,972	903	4,875
Eyes (n)	6,536	1,727	8,263
HVFs (n)	25,723	6,720	32,443
Mean age at first HVF (SD)	62.0 (14.8)	61.8 (15.1)	61.9 (14.8)
Gender			
Male, n (%)	3,026 (46.3%)	768 (44.2%)	3,789 (45.8%)
Female, n (%)	3,510 (53.7%)	969 (55.8%)	4,484 (54.2%)
Eye			
Right, n (%)	3,281 (50.2%)	869 (50.0%)	4,153 (50.2%)
Left, n (%)	3,255 (49.8%)	868 (50.0%)	4,120 (49.8%)
Mean number of HVFs (SD)	3.7 (1.9)	3.6 (1.8)	3.6 (1.9)
Mean follow-up between first and last HVFs in years (SD)	3.5 (2.8)	3.4 (2.8)	3.5 (2.8)
Average mean deviation (SD)	-6.73 (6.26)	-6.70 (6.10)	-6.73 (6.23)

**Fig 2:**
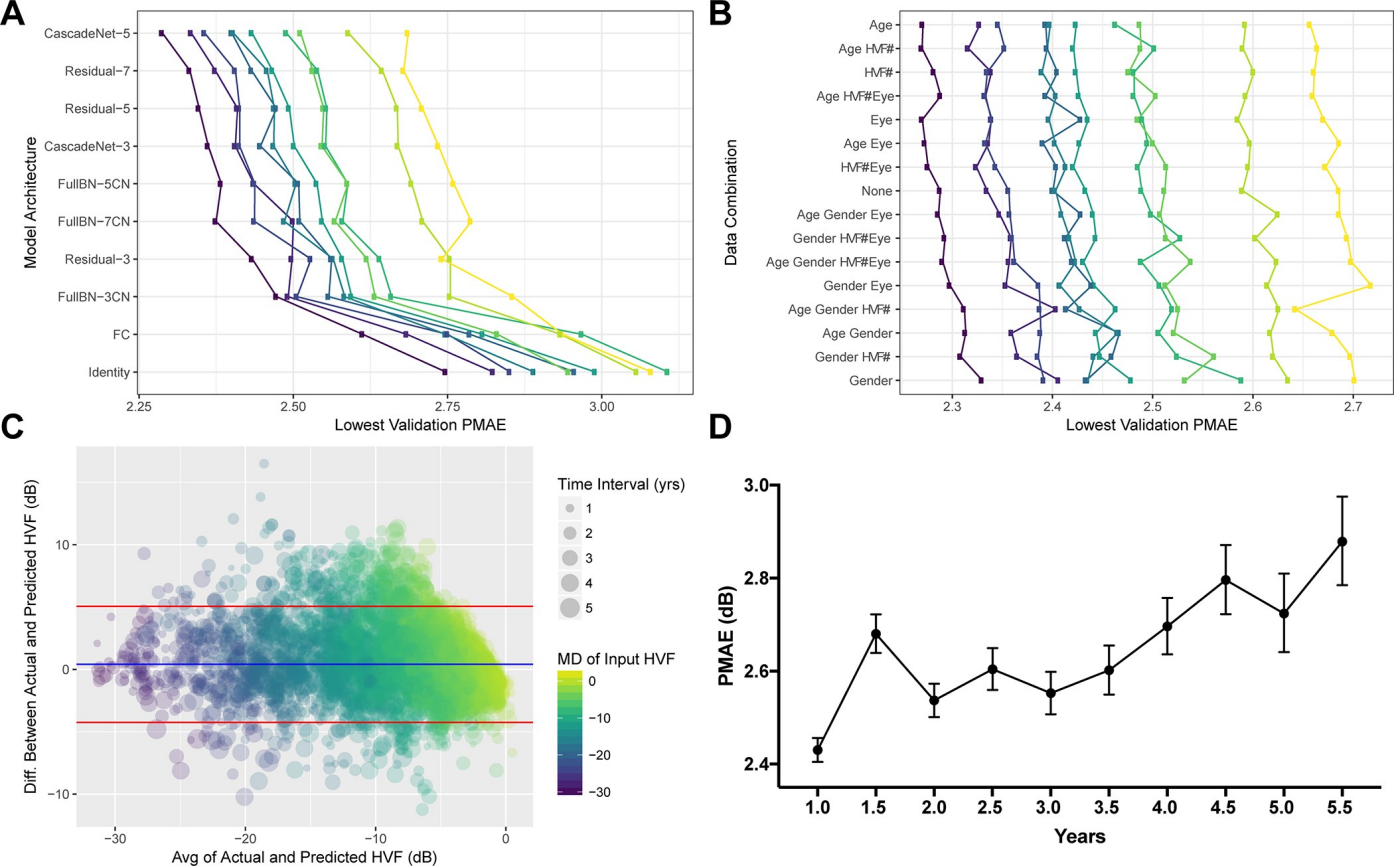
Model development and evaluation. Model development are shown in Panels A and B, Model evaluation on the test set are shown in Panels C and D. A) Model architectures tested with lowest validation Point-wise Mean Absolute Error (PMAE) shown with each colored line representing one of the ten-fold internal cross validation datasets. B) Data combinations tested with every possible combination of age, gender, eye, and test number (HVF #). Model evaluation using held out test set with Bland-Altman plot (C) between Mean Deviation (MD) of the AI predicted and actual future HVFs, (r = 0.92, Adjusted R^2^ = 0.84, p < 2.2 x 10^−16^), color shaded by MD of input (earlier) HVF and sized by time interval. The blue and red lines in (C) represent the mean and 95% CI, respectively. D) Average PMAE for each time interval of held-out test set with 95% confidence intervals as error bars.

Model development occurred in two distinct phases using HVF pairs from 0.75 months to 1.25 months. First, many different model architectures were explored (Table 1). For every model, 10-fold cross validation training was performed and the lowest validation PMAE was recorded. CascadeNet-5 (S2 Fig) resulted in the best network performance compared to the other network tested (Fig 2A).

The second phase of model development was to repeat the test giving the neural networks more clinical context. With the available predictors including age, gender, eye, and the HVF test number (ie the first, second or third, etc. VF test a patient has taken), all 16 possible combinations were trained and the lowest validation PMAE was recorded. Consistently including age as an input into the deep learning model yielded statistically significant superior performance with an improvement of 0.02 dB to the PMAE (p = 0.0003, paired Wilcoxon rank sum test) (Fig 2B). Using CascadeNet-5 and age as an additional input parameter to the first HVF, continuous forward transfer learning was performed to train a separate model for each 0.5 year intervals. The average predictions from final trained models were then used the evaluate the held-out test set.

**Fig 3:**
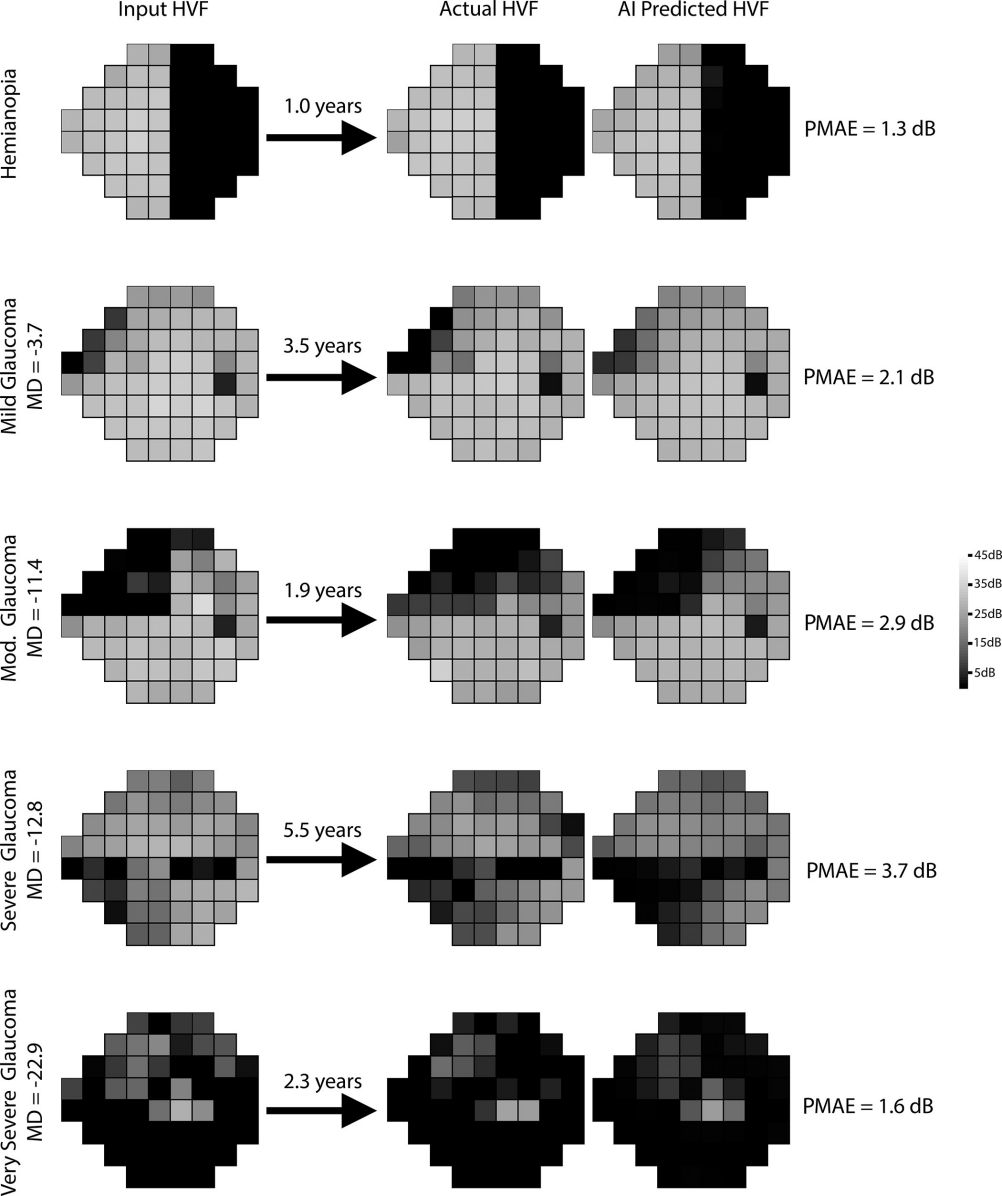
Representative examples from the held-out test set, comparing actual and artificial intelligence (AI) predicted Humphrey Visual Field (HVF). The input HVF is used for predicting HVF at the respective designated time points. A variety of different starting HVFs are shown ranging from a hemifield defect to very severe glaucoma with the corresponding Point-wise Mean Absolute Error (PMAE).

**Fig 4:**
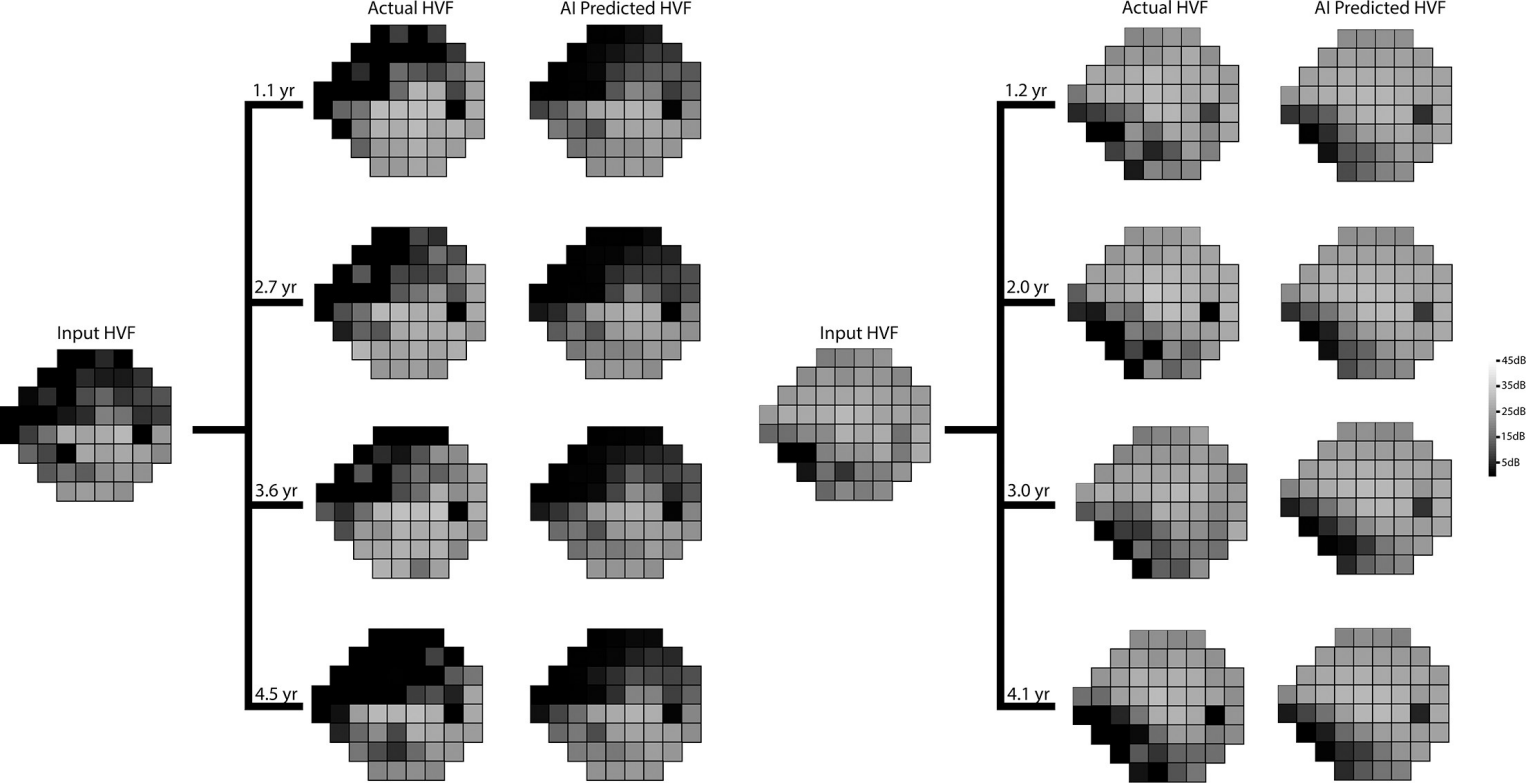
Serial comparisons between actual and artificial intelligence (AI) predicted Humphrey Visual Field (HVF). The single input HVF is used for multiple predictions at different time points. Point-wise mean absolute errors for the left panel from top to bottom were 3.8, 3.8, 5.1, and 4.0 dB respectively, and for the right panel from top to bottom were 2.3, 2.5, 3.2, and 3.2 dB, respectively.

Bland-Altman plot of the difference in mean deviations (Fig 2C) revealed a mean difference of +0.41 dB. A temporal dependence was noted in the prediction accuracy with the model predicting more accurately in shorter time intervals than longer time intervals (Fig 2D). The overall PMAE (Fig 2D) and root mean squared error (RMSE) for the test set were 2.47 dB (95% CI: 2.45 dB to 2.48 dB) and 3.47 dB (95% CI: 3.45 dB to 3.49 dB), using the evaluation strategy shown in S2 Fig. Fig 3 shows representative predictions at different time points against the real HVFs for glaucoma of varying severity, as well as an example of a non-progressing non-glaucomatous hemifield VF defect. Fig 4 shows two examples of serial history in the test set where the same input HVF is used to predict multiple time points in the future.

In comparison to three different linear models, deep learning showed a statistically significant improvement in the PMAE (Table 3). In addition, by relating Jensen’s inequality to the pointwise prediction error, we show that the expected theoretical lower limit of the PMAE for the held-out test set is 2.32 dB (S1 File).

**Table 3:** Held-out test-set performance with deep learning compared to linear models using pointwise mean absolute error (PMAE).

Model	Average PMAE	95% Lower CI	95% Upper CI
Deep learning model	2.47	2.45	2.48
Simulated ROP linear model*	3.77	3.72	3.81
Empirical ROP linear model	3.96	3.91	4.00
Pointwise regressed linear model	3.29	3.24	3.34

Rate of Progression (ROP), Confidence Intervals (CI).

* Based on Early Manifest Glaucoma Trial[25]

## Discussion

Using more than 1.7 million perimetry points from 32,443 consecutive 24–2 HVFs performed during 20 years of routine clinical practice, deep learning was able to predict future VF defects seen in HVFs. The algorithm was able to generate predictions on HVFs at a point-wise level occurring between 0.5 to 5 years. Unsurprisingly, the only clinical factor that modestly improved the AI’s prediction was the patient’s age. The overall PMAE for the test set was 2.47 dB (95% CI: 2.45 dB to 2.48 dB) and the RMSE was 3.47 dB (95% CI: 3.45 dB to 3.49 dB) which represents a statistically significant improvement over linear models (Table 3) and approaches the theoretical lower limit of 2.32 dB.

Prior algorithms for generating predictions of HVFs have centered around fitting regression models for consecutive HVFs and extrapolating the next HVF. Although easy to interpret, simple linear regression analyses such as mean deviation, pattern standard deviation or visual field index do not take into account the spatial nature of visual field loss in glaucoma.[26] Pointwise, exponential regression models have been developed to characterize fast or slow progression rate in VF loss better than linear models.[27] However, these models assume a constant rate of VF deterioration and fail to address varying rates of glaucomatous decay over time.[5] Multiple model approaches that include exponential and logistic function appear to predict future progressions better.[6] Chen et al. reported average RMSEs of 2.925 and 3.056 for logistic and exponential functions, respectively.[6] More recently, a pointwise sigmoid regression model with a mean RMSE of 4.1 has been described to capture the varying rates of glaucomatous deterioration over time and characterize both early and late stages of glaucoma.[5]. Another approach has been in the Bayesian setting to incorporate the spatial data in the VF. Long-term variability in HVF has been one of the major limitations in assessing progression in HVF, and several models have been described to take into account of spatio-temporal correlations in the perimetry data.[28–31] However, the majority of studies were limited by small sample sizes (in the hundreds), data cleaning of the training sets, and evaluation/validation sets with stringent criteria.

A number of recent studies have reported various methods for determining progression among VF series.[32–36] However, few studies attempt to forecast future VFs, and current models that predict future VFs require many VF tests obtained over relatively long periods of time. Using a point-wise ordinary least squares linear regression with series of 15 VFs, Taketani et al. achieved the minimum absolute prediction error of 2.4 ± 0.9 dB using the maximally available number of 14 VFs.[7] As these VFs are acquired approximately every 6 months, many years of testing would be needed to accurately predict future VFs. In another study, Zhu et al. reported the superior performance of analysis with non-stationary Weibull error regression and spatial enhancement (ANSWERS) to permutation of pointwise linear regression for predicting future VF tests.[35] However, even at the shortest prediction interval of 10 months after baseline testing, 6 VFs were used to make the VF prediction.[35] Furthermore, to make a VF prediction for 24 months after baseline, 14 VFs were used.[35] To decrease the number of necessary VFs, Fujino et al. applied a least absolute shrinkage and selection operator regression and reported a prediction error of 2.0 ± 2.2 dB using only 3 VFs.[37] However, even with this model, over a year would need to pass before an accurate prediction could be made. A notable aspect of our approach is that by using deep learning models trained on the temporal history for a large group of patients, we are able to take single HVFs and predict point-wise HVFs at half-year time intervals, up to five years later, with an overall PMAE of 2.57 dB. Prior studies have shown that automated perimetric threshold test variability ranges between 2 and 3 dB for normal subjects[38,39] but can be much more variable in patients with glaucoma.[40] To our knowledge, no prior algorithm has been shown to reliably predict the VF changes based on a single HVF such as ours, to a comparable level of accuracy.

One possible reason for the improved accuracy over prior published models is that our model received an extra 2 digits of precision for each perimetry point. In general, the predicted HVFs tended to be slightly less progressed than the actual HVFs, as seen in the Bland-Altman plot (Fig 2C), whereby the mean difference in MD between predicted and actual HVFs was 0.41 dB. This may be because the dataset we used to train our algorithm was unfiltered. Our dataset included non-glaucomatous VFs and VFs after ocular surgeries that may increase perimetry sensitivity, such as cataract extraction. The majority of studies on forecasting VFs and estimating VF progression utilize highly filtered datasets with strict inclusion and exclusion criteria. Most require a certain number of VF tests per study subject that meet certain reliability criteria, and/or a known diagnosis of glaucoma before inclusion. Our model was developed using unfiltered data and included every single VF test performed at our institution regardless of diagnosis or test reliability, and yet the algorithm was still able to make reasonably accurate predictions. Even in cases of non-glaucomatous VF changes, such as a hemifield defect from a cerebrovascular accident (Fig 3), the program forecasts an appropriate future VF, which in this example is a stable VF defect, likely based on the predicted stability of both unaffected and severely affected points. Conversely, the VF for a moderate glaucoma example in Fig 3 has the appearance of a quadrantanopia on cursory visual inspection, yet the program was able to accurately predict that it would progress in a glaucomatous manner. The algorithm would likely perform even better were the program trained and evaluated on a rigorously filtered dataset.

Previous applications of deep learning in glaucoma have been limited to classification rather than forecasting and included analysis of disc photos,[17,19,41] OCT images,[18,42] and VFs.[15] Recently, Li et al. used a deep learning system to classify over 48,000 optic disc photos for referable glaucomatous optic neuropathy and reported an area under the receiver operating characteristic curve (AUROC) of 0.986 with a 95.6% sensitivity and 92.0% specificity.[19] Using a hybrid deep learning method to analyze 102 single wide-field OCT scans, Muhammad et al. found that their protocol outperformed standard OCT and VF clinical metrics in differentiating suspects from early glaucomatous eyes.[18] Asaoka et al. used a multilayer, feed-forward neural network to detect preperimetric glaucoma VFs (AUC 92.6%, 95% CI 89.8–95.4) using 171 preperimetric VFs and 108 control VFs.[15] The deep learning classifier performed significantly better than other machine learning algorithms in distinguishing preperimetric glaucoma from healthy VFs. Unlike these previous classification studies, our study demonstrates a novel application of deep learning to build generative models in glaucoma.

Ideally, forecasted VFs would guide clinical decision-making and disease management. Kalman Filter models have been recently developed to determine the best VF testing frequency to detect glaucoma progression most efficiently and also to forecast glaucoma progression trajectories at different target intraocular pressures.[43,44] Our current model incorporates very little clinical data, although the predictive accuracy was already improved after including subject age. Future research will focus on developing models that incorporate additional clinical parameters, such as IOP, medication and surgical intervention, to assess their effects on forecasted VFs and progression, which may aid in clinical decision making. Additionally, our model could be used to determine how HVFs would change depending on the intervention selected for each individual patient. This type of AI application may help enable precision medicine for glaucoma management in the future.

Our study has several limitations. First, this study included data from a single academic center and only included 24–2 HVF tests, thus our AI-algorithm may not be widely applicable. Furthermore, because the patients included in this study were actively being treated for their respective diseases, predictions may vary with management. However, we included all available 24–2 HVFs over a twenty year period, including numerous providers of different subspecialties, with no filtering, to improve the generalizability of the algorithm. Future work may include training with datasets from multiple centers and testing in a geographically separated cohort. Second, our test set did not include any pairs of HVFs that were beyond 5.5 years of duration. With this current AI-algorithm, predictions beyond 5.5 years of duration became less accurate. Future algorithms that incorporate more clinical parameters will hopefully produce accurate predictions for greater future durations. It is also possible that longitudinal data of longer duration (i.e. >5.5 years) may have included more HVFs with progression to advanced glaucomatous loss resulting in substantial change in the decay rate. Under such circumstances, e.g. for the rapidly progressing glaucoma patient, the AI-algorithm may be less accurate in its predictions given the unusual and less common progression pattern. Third, given the variability of VF testing, it is possible for the AI-algorithm to be misled by a poor baseline VF test due to learning effects. In the Ocular Hypertension Treatment Study, 85.9% of VF abnormalities in reliable tests were not confirmed on retesting.[45] Indeed, upon review of our top outliers, a number of them resulted from a baseline VF demonstrating various defects that led the AI-algorithm to forecast a severely depressed future field, while the actual VF paradoxically improved considerably. Finally, the AI-algorithm was not trained as a classification tool; rather, it was constructed to generate prediction maps for future HVFs that incorporate both spatial and temporal data, a more challenging task for AI than the classification of a subject as progressed or stable.

In conclusion, we have developed a novel deep learning algorithm that is able to forecast, with reasonable accuracy, HVFs up to 5 years from a single HVF as input. The ability to quickly anticipate future glaucomatous progression may prevent unnecessary functional loss that can occur with the current practice of multiple confirmatory tests. With further incorporation of clinical data such as IOP, medication and surgical history, our AI-model may assist in clinical decision-making in the future and allow development of a personalized treatment regimen for each patient.

## Supporting information

S1 Fig Evaluation strategy for test set after models training. For each of the ten time intervals, there are 10 trained models, one for each cross validation set. Each HVF prediction is then averaged together and the final prediction is compared with the actual HVF.
(PDF)

S2 Fig Simplified schematic of the CascadeNet architecture. CascadeNet-1 has 1 copy of convolutional (CN) layers of 64, 128, and 256 filters. CascadeNet-2 has 2 copies of CN layers of 64, 128, and 256 filters. Each black arrow represents a copy-concatenation of the output tensors “cascaded” forward to every possible neural network block. The final model, CascadeNet-5 has 5 copies of CN layers with 64, 128, and 256 filters.
(PDF)

S1 File Mathematical modeling description for HVF progression. Baseline models are described in depth as well as a derivation of the theoretical limit of point-wise prediction models.
(PDF)
