# Supplementary material for: Forecasting future Humphrey Visual Fields using deep learning
Source: PLoS One. 2019 Apr 5;14(4):e0214875. doi: 10.1371/journal.pone.0214875 (PMC6450620; doi:10.1371/journal.pone.0214875)
Supplement: S1 Fig — For each of the ten time intervals, there are 10 trained models, one for each cross validation set. Each HVF prediction is then averaged together and the final prediction is compared with the actual HVF. (PDF) [file pone.0214875.s001.pdf]

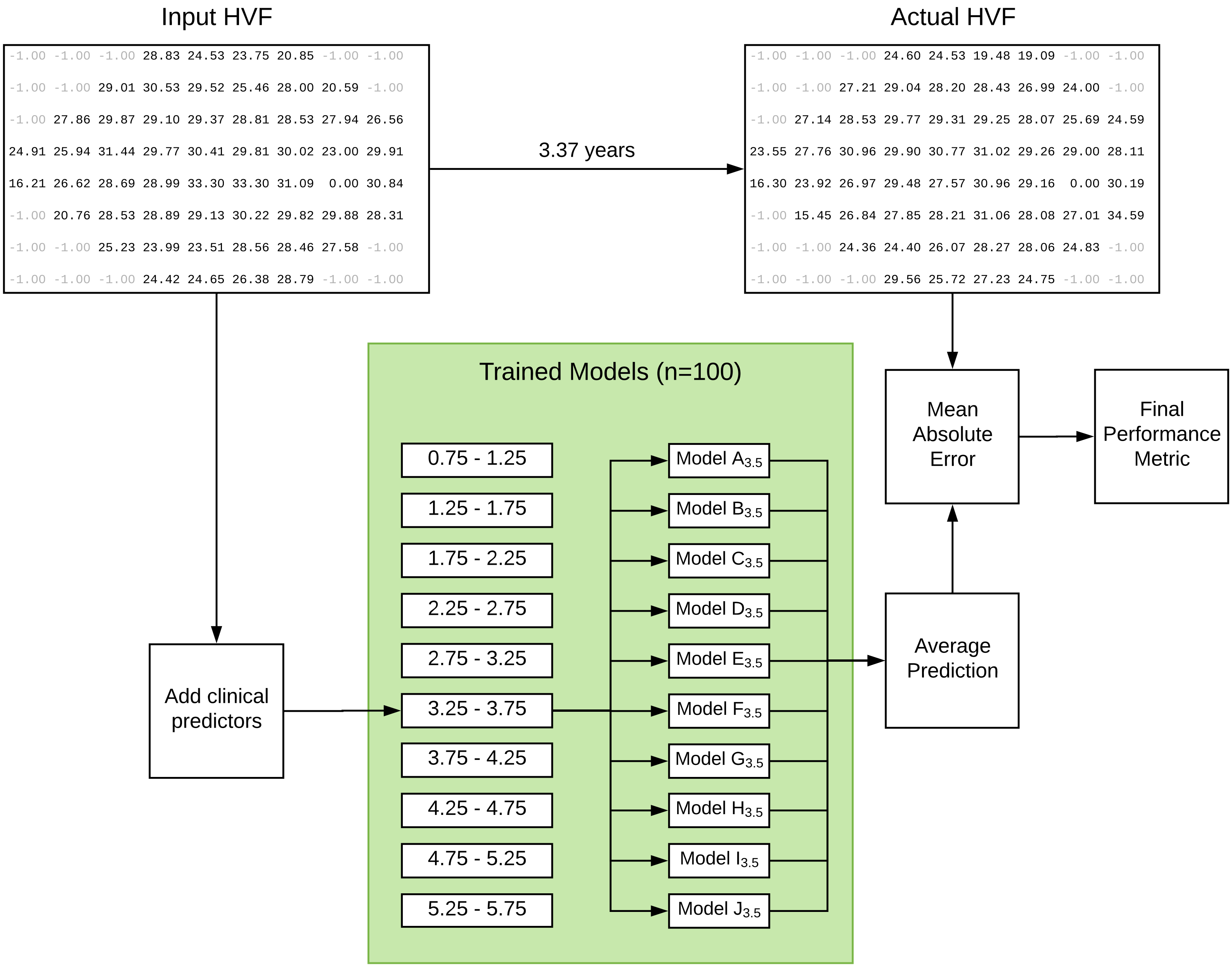

Supplementary Figure 1: Evaluation strategy for test set after models training. For each of the ten time interval, there are 10 trained models, one for each cross validation set. Each HVF prediction is then averaged together and the final prediction is compared with the actual HVF.
