# Supplementary material for: Forecasting future Humphrey Visual Fields using deep learning
Source: PLoS One. 2019 Apr 5;14(4):e0214875. doi: 10.1371/journal.pone.0214875 (PMC6450620; doi:10.1371/journal.pone.0214875)
Supplement: S2 Fig — CascadeNet-1 has 1 copy of convolutional (CN) layers of 64, 128, and 256 filters. CascadeNet-2 has 2 copies of CN layers of 64, 128, and 256 filters. Each black arrow represents a copy-concatenation of the output tensors “cascaded” forward to every possible neural network block. The final model, CascadeNet-5 has 5 copies of CN layers with 64, 128, and 256 filters. (PDF) [file pone.0214875.s002.pdf]

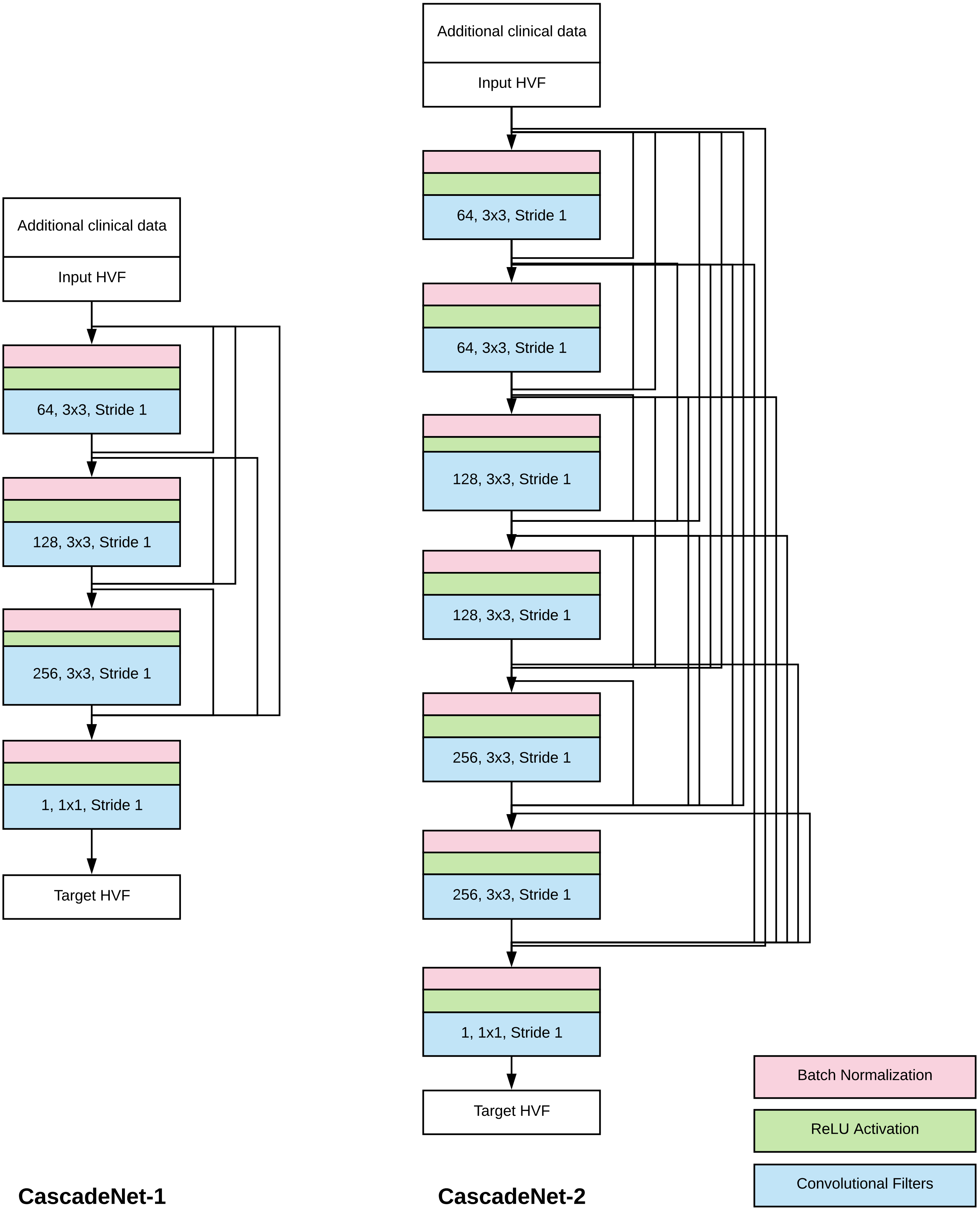

Supplementary Figure 2: Simplified schematic of the CascadeNet architecture. CascadeNet-1 has 1 copy of convolutional (CN) layers of 64, 128, and 256 filters. CascadeNet-2 has 2 copies of CN layers of 64, 128, and 256 filters. Each black arrow represents a copy-concatenation of the output tensors “cascaded” forward to every possible neural network block. The final model, CascadeNet-5 has 5 copies of CN layers with 64, 128, and 256 filters.
