# Supplementary material for: Forecasting future Humphrey Visual Fields using deep learning
Source: PLoS One. 2019 Apr 5;14(4):e0214875. doi: 10.1371/journal.pone.0214875 (PMC6450620; doi:10.1371/journal.pone.0214875)
Supplement: S1 File — Baseline models are described in depth as well as a derivation of the theoretical limit of point-wise prediction models. (PDF) [file pone.0214875.s003.pdf]

Supplementary Material

Yue Wu

1 Introduction

This supplementary document provides a discussion of the deep learning model along with the baseline models in Section 3. Then in Section 4, we demonstrate why predicting point-wise Humphrey Visual Fields is a much harder task than predicting the mean HVF. Finally, we derive some theoretical limits for point-wise HVF predictions in Section 5.

2 Notation

Before we start, we will describe our data and clarify our notation. Our data consists of 38,964 and 8,962 paired point-wise HVFs in the training and test datasets, respectively. Each HVF has up to 54 perimetry measurements because of the HVF 24-2 SITA acquisition protocol. For notational clarity, let $\{X_{0i}^n\}_{i=1}^{54}$ and $\{X_{t_n i}^n\}_{i=1}^{54}$ denote the starting and end point-wise HVFs for the nth paired HVFs in the training data. Then $X_{t_n i}$ represents the ith perimetry point, measured in decibels (dB) of sensitivity, for the nth end HVF. It is clear that each set has 54 point-wise measurements, and that $t_n > 0$ represents the time-difference between the start and end HVF in the nth pair.

Furthermore, to differentiate test data from training data, we adopt the same notation, but use Y instead of X. Next, we use the common convention of $\hat{X}$ to denote a predicted value of X.

3 Models

All the models, the deep learning and baseline models, can be expressed as

$$\hat{Y}_{t_n i} = f(Y_{0i}, \{Y_{0i}\}_{i=1}^{54}, t_n, \theta) \tag{1}$$

Here $\hat{Y}_{t_n i}$ denotes the prediction for the ith perimetry point of the nth paired data in the test data, given time between paired observations of t_n, initial measured perimetry Y_{0i}, the other perimetry points $\{Y_{0i}\}_{i=1}^{54}$, which may provide predictive power through the local structure of the Humphrey Visual field, and parameters θ learnt on the training data $\{X\}$ or external data. The main differences between the models are in the functional form of f and the role of the function parameters $(Y_{0i}, \{Y_{0i}\}_{i=1}^{54}, t_n, \theta)$.

3.1 Simulated ROP Model

The simplest point-wise prediction model is the ROP model, which can be written as:

$$E[\hat{Y}_{t_n i}] = Y_{0i} + \beta \cdot t_n \tag{2}$$

$$Var[\hat{Y}_{t_n i}] = \sigma^2 \cdot t_n^2 \tag{3}$$

$$\hat{Y}_{t_n i} \sim \mathbb{N}(Y_{0i} + \beta \cdot t_n, \sigma \cdot t_n) \tag{4}$$

Here $\theta = \{\beta, \sigma\}$, where β and σ are the mean and standard deviation of progression per year, and f is a linear function in Y_{0i}. When written in this way, this baseline model is clearly a linear Gaussian model. It is linear in the mean progression of the ROP (2), with the noise modeled as a Gaussian distribution with standard deviation $\sigma \cdot t_n$ (4).

Now for our first baseline, we took (4), and used $\beta^{(g)} = -0.36$ dB / year and $\sigma^{(g)} = 0.60$ dB / year, which were determined in the Early Manifest Glaucoma Trial. For notational clarity, we adopt the superscript (g) to show that these parameters came from a general study of glaucoma patients rather than the exponential operation, and to differentiate from our second baseline.

3.2 Empirical ROP Model

The empirical ROP model shares the same functional form as the ROP model (4). The only difference is that instead of using $\beta^{(g)}$ and $\sigma^{(g)}$ from the Early Manifest Glaucoma Trial, we use the empirical $\beta^{(e)}$ and $\sigma^{(e)}$ measured on our training dataset. Specifically, we computed the mean and standard deviation of the time-adjusted change for all point-wise HVFs, $[\{\frac{1}{t_n}(X^n_{t_n i} - X^n_{0i})\}^{54}_{i=1}]^{N=38964}_{n=1}$ for the 38,964 paired observations in our training data.

The motivation for the empirical ROP model is that our dataset may be internally consistent, but different from the data in the Early Manifest Glaucoma Trial due to differing baseline real-world patient characteristics and the stringent inclusion and exclusion criteria used in the trials.

3.3 Regressed Pointwise Model

The regression model is a generalization of the ROP Model since it can be expressed as:

$$\hat{Y}_{t_n i} \sim \mathbb{N}(Y_{0i} + \beta_i \cdot t_n, \sigma_i \cdot t_n) \tag{5}$$

Here the parameters β_i and σ_i are indexed so that each perimetry point has its own rate of progression parameters. These individual progression parameters were found by regressing the change in $\{X^n_{t_n i} - X^n_{0i}\}^{N=38964}_{n=1}$ vs $\{t_n\}^{N=38964}_{n=1}$ for each i separately in the training data. Table 1 shows regressed coefficients. Compared to (4), there are $160 = 54 \cdot 3 - 2$ more degrees of freedom, since each regression has an intercept, slope and standard deviation.

Slope									Intercept								
			-0.338	-0.339	-0.365	-0.384						0.277	0.305	0.403	0.503		
		-0.312	-0.267	-0.288	-0.326	-0.313	-0.337				0.222	0.161	0.191	0.299	0.299	0.361	
	-0.317	-0.308	-0.289	-0.279	-0.352	-0.335	-0.321	-0.312		0.212	0.246	0.226	0.178	0.316	0.311	0.322	0.336
-0.404	-0.361	-0.317	-0.335	-0.350	-0.287	-0.341	-0.330	-0.269	0.389	0.296	0.179	0.228	0.167	0.218	0.367	0.338	0.295
-0.417	-0.357	-0.330	-0.314	-0.325	-0.254	-0.331		-0.275	0.360	0.269	0.15	0.119	0.144	0.162	0.281		0.270
	-0.376	-0.296	-0.325	-0.343	-0.362	-0.338	-0.315	-0.266		0.298	0.172	0.136	0.116	0.172	0.212	0.344	0.222
		-0.36	-0.296	-0.307	-0.287	-0.296	-0.356				0.213	0.153	0.125	0.185	0.208	0.382	
			-0.346	-0.331	-0.303	-0.355						0.295	0.297	0.203	0.312		

Table 1: Parameters for regressed pointwise model

3.4 Deep Learning Model

The baseline models are simplistic models that only consider linear rate of progression for the HVF. Moreover in these baseline models, the perimetry points were considered independently of each other. Clearly this does not leverage local HVF structure where one would expect neighbouring perimetry values to be correlated to each other, with the correlation highest for neighbouring points and lower for points more distant from each other. The deep learning model is able to capture this structure since its function is the most general form (1). Moreover, not only does it take into account neighbouring points, it also naturally fits non-linear functions to all this data to make point-wise predictions.

4 Theoretical limits of point-wise prediction models

This is one of the first papers to predict point-wise HVFs. We note that making point-wise predictions are much harder than predicting the mean HVF. Let the metric for prediction accuracy be the mean absolute error (MAE). Then the point-wise MAE (PMAE) for predictions on the test dataset can be expressed as:

$$\begin{aligned} PMAE &= \frac{1}{N}\sum_{n=1}^{N}\frac{1}{54}\sum_{i=1}^{54}|\hat{Y}^n_{t_n i} - Y^n_{t_n i}| \\ &= \frac{1}{N}\frac{1}{54}\sum_{n=1}^{N}\sum_{i=1}^{54}|\hat{Y}^n_{t_n i} - Y^n_{t_n i}| \end{aligned} \tag{6}$$

On the other hand the MAE for the mean HVF is:

$$\begin{aligned} MMAE &= \frac{1}{N}\sum_{n=1}^{N}\left|\left(\frac{1}{54}\sum_{i=1}^{54}\hat{Y}^n_{t_n i}\right) - \left(\frac{1}{54}\sum_{i=1}^{54}Y^n_{t_n i}\right)\right| \\ &= \frac{1}{N}\sum_{n=1}^{N}\left|\frac{1}{54}\sum_{i=1}^{54}(\hat{Y}^n_{t_n i} - Y^n_{t_n i})\right| \\ &= \frac{1}{N}\frac{1}{54}\sum_{n=1}^{N}\left|\sum_{i=1}^{54}(\hat{Y}^n_{t_n i} - Y^n_{t_n i})\right| \end{aligned} \tag{7}$$

Then by Jensen's Inequality:

$$\frac{1}{N}\frac{1}{54}\sum_{n=1}^{N}\left|\sum_{i=1}^{54}(\hat{Y}^n_{t_n i} - Y^n_{t_n i})\right| \leq \frac{1}{N}\frac{1}{54}\sum_{n=1}^{N}\sum_{i=1}^{54}|\hat{Y}^n_{t_n i} - Y^n_{t_n i}| \tag{8}$$
$$\implies MMAE \leq PMAE$$

The equality condition is only reached if and only if $(\hat{Y}^n_{t_n i} - Y^n_{t_n i}) = |\hat{Y}^n_{t_n i} - Y^n_{t_n i}|$ for every term. Therefore the MMAE for the predicted mean HVF is a lower limit for the PMAE of the point-wise HVF predictions.

4.1 Relationship of MMAE to change in mean deviation

The MMAE may be more familiar to clinicians as the mean predicted change in mean deviation (MD). To show this, we first express the mean deviation in our notation. For patient n, the mean deviation is:

$$MD^n = \frac{1}{54}\sum_{i=1}^{54}(Y^n_i - a_i), \tag{9}$$

where a_i is the population age-adjusted standard for perimetry point i. Then the change in MD over time t_n for patient n, with a_i cancelling each other, is:

$$\Delta MD^n_{t_n} = MD^n_{t_n} - MD^n_0 \tag{10}$$

This is the change in MD for realized observations. The MMAE is the mean predicted change in MD since it is $\hat{MD}^n_{t_n}$ instead of $MD^n_{t_n}$ in Equation (10), and it is averaged over all patients:

$$MMAE \propto \frac{1}{N}\sum_{n=1}^{N}(\hat{MD}^n_{t_n} - MD^n_{t_n}) = \frac{1}{N}\sum_{n=1}^{N}\frac{1}{54}\sum_{i=1}^{54}(\hat{Y}^n_{t_n i} - Y^n_{t_n i}) \tag{11}$$

5 Measuring lower limit for point-wise prediction models

Having established that the MMAE is the lower limit of the PMAE, we proceed to determine the value of this limit. Obviously the limit will be the smallest MMAE for the test data. Since the MMAE is the mean of the absolute difference in predicted mean vs observed mean HVFs, it depends on how accurately the mean HVF is predicted on the test data. A natural model for predicting mean HVF is:

$$\hat{\bar{Y}}_{t_n} \sim \mathbb{N}(\bar{Y}_0 + \beta \cdot t_n, \sigma \cdot t_n) \tag{12}$$

This is similar to Equation (4), except here mean $\bar{Y}_{t_n}$ is being predicted.

Then given this mean HVF prediction model (12), the lowest MMAE is determined by finding the optimal rate of progression parameter, β^{opt} on the test data. Specifically, we gridded candidate β from $-1, -.99, -.98, ..., .99, 1$. The standard deviation was fixed to be the standard deviation of the test data, $\sigma = 0.56$. Then predicted using Equation (12), and computed the MMAE with Equation (7).

The optimal β^{opt} was found to be -0.17. This yields a MMAE of 2.32 dB, which is the lower limit for the PMAE. For comparison, the PMAE of our deep learning method was 2.45 dB. In contrast, the PMAE of the baseline linear models were 3.77 dB, 3.96 dB and 3.29 dB. This shows that our deep learning predictions approach this lower limit.
